# Association between *MANBA* Gene Variants and Chronic Kidney Disease in a Korean Population

**DOI:** 10.3390/jcm10112255

**Published:** 2021-05-23

**Authors:** Hye-Rim Kim, Hyun-Seok Jin, Yong-Bin Eom

**Affiliations:** 1 Department of Medical Sciences, Graduate School, Soonchunhyang University, Asan 31538, Chungnam, Korea; goa6471@naver.com; 2 Department of Biomedical Laboratory Science, College of Life and Health Sciences, Hoseo University, Asan 31499, Chungnam, Korea; jinhs@hoseo.edu; 3 Department of Biomedical Laboratory Science, College of Medical Sciences, Soonchunhyang University, Asan 31538, Chungnam, Korea

**Keywords:** *MANBA*, variants, chronic kidney disease, estimated glomerular filtration rate (eGFR), expression quantitative trait loci (eQTL)

## Abstract

Chronic kidney disease (CKD), a damaged condition of the kidneys, is a global public health problem that can be caused by diabetes, hypertension, and other disorders. Recently, the *MANBA* gene was identified in CKD by integrating CKD-related variants and kidney expression quantitative trait loci (eQTL) data. This study evaluated the effects of *MANBA* gene variants on CKD and kidney function-related traits using a Korean cohort. We also analyzed the association of *MANBA* gene variants with kidney-related traits such as the estimated glomerular filtration rate (eGFR), and blood urea nitrogen (BUN), creatinine, and uric acid levels using linear regression analysis. As a result, 14 single nucleotide polymorphisms (SNPs) were replicated in CKD (*p* < 0.05), consistent with previous studies. Among them, rs4496586, which was the most significant for CKD and kidney function-related traits, was associated with a decreased CKD risk in participants with the homozygous minor allele (CC), increased eGFR, and decreased creatinine and uric acid concentrations. Furthermore, the association analysis between the rs4496586 genotype and *MANBA* gene expression in human tubules and glomeruli showed high *MANBA* gene expression in the minor allele carriers. In conclusion, this study demonstrated that *MANBA* gene variants were associated with CKD and kidney function-related traits in a Korean cohort.

## 1. Introduction

Chronic kidney disease (CKD) is an important health problem worldwide and increases mortality and morbidity by raising the risk of several diseases [1,2]. According to the Global Burden of Disease (GBD) study, the global prevalence of CKD in 2017 was estimated at 9.7%, with approximately 697.5 million people affected [3]. The prevalence of CKD in Korea in 2017 was 3% according to the chronic disease health statistics of the Korea Disease Control and Prevention Agency (KDCA) (https://health.cdc.go.kr/ (accessed on 13 April 2021)). In addition, the GBD study reported that fatality from CKD in 2017 was 1.2 million, the 12th primary cause of death worldwide. The common causes of impaired kidney function are diabetes, hypertension, and glomerulonephritis, which can be evaluated by the estimated glomerular filtration rate (eGFR) [4]. Previous studies have estimated the heritability of CKD to be 20–80% by measuring the contribution of genetic effects in a population of patients with CKD [5,6,7]. Therefore, since CKD is also affected by genetic factors, it is important to identify the risk factors associated with CKD development and genes in individuals with CKD.

A meta-analysis of genome-wide association studies (GWAS) on CKD and kidney function-related traits was performed in European, Asian, and African populations [8,9,10]. Previous studies have determined non-coding genetic variants related to CKD through GWAS but the elucidation of the underlying genes and mechanisms was limited. Therefore, a recent study performed expression quantitative trait loci (eQTL) analysis of renal glomeruli and tubular tissue and found 27 candidate genes that could be potential causes of kidney disease development [11]. Among them, *MANBA*, *PGAP3,* and *CASP9* were confirmed to have functional roles in kidney disease development in previous animal studies [12,13,14]. Another study reported that variants of the *MANBA* gene identified using eQTL analysis and CKD-related variants identified using GWAS analysis showed statistically significant co-localization [12]. In addition, Gu et al. demonstrated the in vivo mechanism by which the *MANBA* gene affects kidney disease [15].

The *β*-mannosidase (*MANBA*) gene, encoding lysosome *β*-mannosidase, is located on human chromosome 4q24 [16,17]. Although many genetic variants associated with CKD have been identified, correlation analysis focusing on *MANBA* gene variants that directly affect kidney diseases are rare. Therefore, in this study, the association analysis of *MANBA* gene variants with CKD and kidney function-related traits was performed in the Korean Genome and Epidemiology Study (KoGES) cohort. We found 20 single nucleotide polymorphisms (SNPs) that showed a statistically significant association with CKD and kidney function-related traits among 229 SNPs of the *MANBA* gene. In addition, rs4496586, which had the highest significance for CKD, was associated with *MANBA* gene expression in renal tubules and glomeruli. These results replicate previous studies that functionally demonstrated the association between kidney disease and the *MANBA* gene.

## 2. Materials and Methods

### 2.1. Participants

The epidemiological data used in this study were obtained from the Health Examinee (HEXA) cohort of the Korean Genome and Epidemiology Study (KoGES). From 2004 to 2013, a total of 173,208 participants aged over 40 years were recruited in the HEXA cohort. Among them, genotype data on 58,700 participants were available. A more detailed description of the HEXA cohort has been described previously [18]. The Institutional Review Board (IRB) of the Korea Disease Control and Prevention Agency (KDCA, KBN-2021-003 (26 January 2021)) and Soonchunhyang University (202012-BR-086-01 (15 December 2020)) approved the study protocol. Written informed consent was obtained from all subjects. All methods were performed in accordance with the relevant guidelines and regulations.

### 2.2. Basic Characteristics

The parameters measured in this study included physical measurements such as height, and weight, and biochemical measurements such as uric acid, blood urea nitrogen (BUN), and serum creatinine levels. The characteristics of the participants in this study are shown in Table 1. Body mass index (BMI) was calculated by dividing body weight (kg) by height squared (m^2^). Blood samples were collected after an 8-h fast and all plasma samples were measured biochemically. Serum creatinine concentrations were measured by the Jaffe method using an automatic analyzer (Hitachi, Tokyo, Japan).

### 2.3. Definition of Chronic Kidney Disease

For the case-control analysis of CKD, control groups (30,813 participants) and cases (1130 participants) were classified according to the recommendations of the Kidney Disease Improving Global Outcome (KDIGO) guidelines. CKD was defined as an eGFR of <60 mL/min/1.72 m^2^ and a history of renal disease. Non-CKD was defined as an eGFR of ≥90 mL/min/1.72 m^2^. Known as the best estimate of kidney function, the eGFR was calculated through the creatinine-based modification of diet in renal disease (MDRD) equation. Kidney function was additionally assessed by BUN, uric acid, and serum creatinine levels.

### 2.4. Genotyping

The genotype data were provided by the Center for Genome Science, Korea National Institute of Health. A total of 58,700 genomic DNA samples isolated from peripheral blood were genotyped using the Affymetrix Axiom^®^ Array (Affymetrix, Santa Clara, CA, USA). The genotype data were confirmed using the Korean-Chip (K-CHIP, Seoul, Korea) acquired by the K-CHIP consortium. Detailed information on Korean chips has been described previously [19]. Samples with less than 96%–99% genotyping accuracy, excessive heterozygosity, or sex inconsistency were excluded. Markers with missing genotype rates of <95%, a minor allele frequency of <1%, and Hardy-Weinberg equilibrium *p*-values of <1 × 10^−6^ were also excluded to control the quality of the genotyping results. After quality control, imputation analysis was performed using the 1000 genome phase 3 dataset (reference panel) in IMPUTE v2 software. A total of 8,056,211 SNPs were included in the study. This study selected SNPs significantly related to kidney disease in the *MANBA* gene. The location of the SNPs was identified using National Center for Biotechnology Information (NCBI) Human Genome Build 37 (hg19).

### 2.5. Statistical Analysis

Statistical analyses were conducted with PLINK version 1.90 beta (https://www.cog-genomics.org/plink2 (accessed on 13 April 2021)) [20] and PASW Statistics version 18.0 (SPSS Inc. Chicago, IL, USA). A total of 58,700 participants were classified as CKD cases and controls according to KDIGO guidelines, and logistic regression analysis was performed to calculate the odds ratios (ORs) and 95% confidence intervals (95% CIs). Linear regression analysis was used to analyze the association between BUN, uric acid, and creatinine levels, and eGFR, which are traits related to kidney function, and *MANBA* genes. Logistic and linear regression analyses were performed based on the additive genetic model after age and gender adjustments. The significance threshold (*p* < 6.76 × 10^−4^) was adjusted through Bonferroni correction. Regional plots were created using the LocusZoom program (http://locuszoom.org/ (accessed on 13 April 2021)). A conditional analysis was performed to identify secondary association signals. The publicly available Human Kidney eQTL database (http://susztaklab.com/eqtl (accessed on 13 April 2021)) was used to determine whether the variants significantly associated with CKD affected the expression level of the *MANBA* gene.

## 3. Results

### 3.1. Participant Characteristics

The clinical characteristics of the 58,700 participants (20,293 men, and 38,407 women) in this study are shown in Table 1. To analyze the association between CKD and variants in the *MANBA* gene, CKD was divided into cases and controls using the eGFR. BUN, uric acid, and creatinine levels, which are related to kidney function, were increased in the case group compared to the control group. The comparison between the CKD cases and controls using the Student’s *t*-test showed that all characteristics except BMI were significantly different.

### 3.2. Association Analysis of the MANBA Gene Variants with CKD and Kidney Function-Related Traits

The present study investigated the association of 229 SNPs in the *MANBA* gene with CKD and kidney function-related traits such as eGFR, BUN, creatinine, and uric acid levels. Before analyzing the association, we selected SNPs that tagged other SNPs with *r*^2^ < 0.8 to show the independently related SNPs of the *MANBA* gene. As a result, 74 of 229 SNPs were identified, and finally, 20 SNPs achieved significance of *p* < 6.76 × 10^−4^ (Bonferroni threshold: *p* = 0.05/74) for association with CKD or kidney function-related traits in the HEXA cohort (Table 2). Most of the 20 SNPs are significantly associated with kidney function-related traits such as eGFR, creatinine and uric acid levels. However, there was no association between SNPs in the *MANBA* gene and BUN levels. The odds ratio of CKD was 0.87 (95% CI 0.80–0.95, *p* = 2.61 × 10^−3^) for the rs4496586 which showed the highest association with CKD. The linear regression analysis showed a significant association of rs4496586with eGFR (*β* = 0.565, *p* = 1.43 × 10^−9^), creatinine (*β* = −0.004, *p* = 1.04 × 10^−8^), and uric acid levels (*β* = −0.019, *p* = 1.83 × 10^−3^). These results were consistent with the reduced risk of CKD in patients carrying the minor C allele of rs4496586.

**Figure 1:**
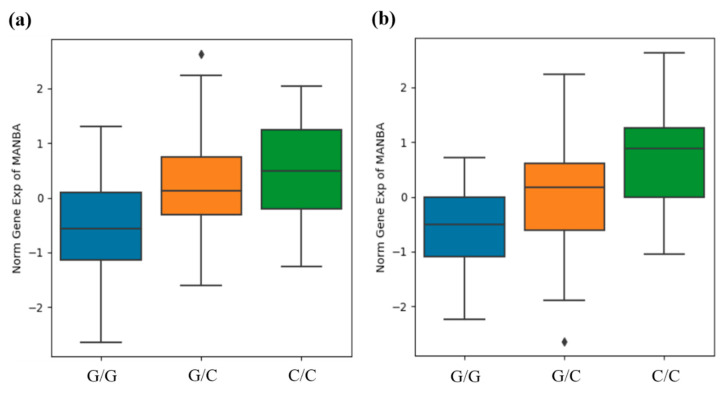
Rs4496586 genotype and *MANBA* gene expression association in human kidney tubules (**a**) and glomeruli (**b**). The data are from the Human Kidney eQTL database (http://susztaklab.com/eqtl (accessed on 13 April 2021)). *MANBA* gene expression for the rs4496586 genotype in tubules (*β* = 0.636, *p* = 1.44 × 10^−6^) and glomeruli (*β* = 0.609, *p* = 2.49 × 10^−6^) was confirmed and statistically significant. *p*-value was calculated by linear regression. Center lines show medians, box limits indicate 25th and 75th percentiles, and whiskers extend to the 5th and 95th percentiles, outliers are represented by diamonds.

Additionally, the Human Kidney eQTL database was used to analyze the association between the rs4496586 genotype, which had a high significance for CKD, and the *MANBA* gene expression in the renal tubules and glomeruli. The expression level of the *MANBA* gene was significantly increased in patients with the minor C allele of rs4496586 in the renal tubules and glomeruli (Figure 1).

**Figure 2:**
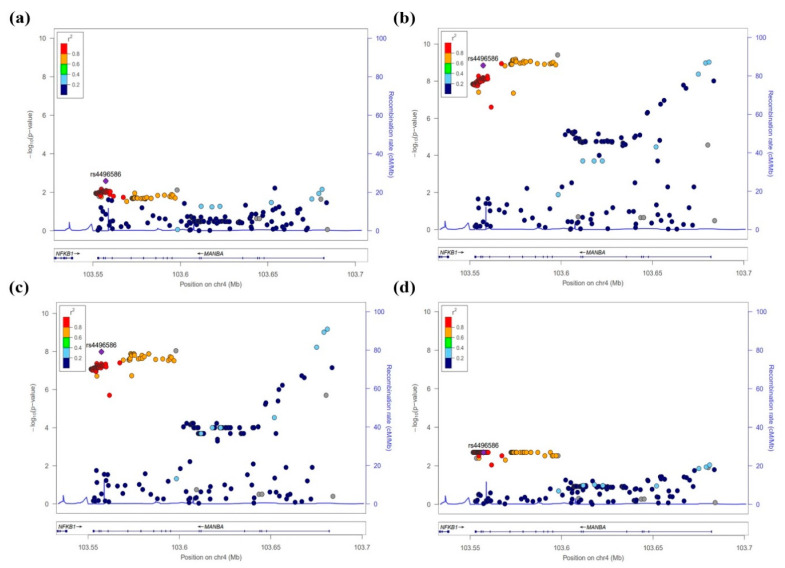
Regional plots of the association results of *MANBA* SNPs with CKD (**a**) and kidney function-related traits such as eGFR (**b**), creatinine (**c**), and uric acid levels (**d**) in the HEXA cohort. The statistical significance (−log_10_ *p*-value) of the analyzed SNPs is plotted. The colors indicate the linkage disequilibrium (*r*^2^) between rs4496586 and the remaining SNPs. The genetic recombination rates are shown on the right *y*-axis. The plots were generated by the LocusZoom program (http://csg.sph.umich.edu/locuszoom (accessed on 13 April 2021)).

The SNPs of the *MANBA* gene, which were significant in the association analysis for CKD and kidney function-related traits, were shown using LocusZoom (http://csg.sph.umich.edu/locuszoom/ (accessed on 13 April 2021)), confirming the regional plots (Figure 2, Supplementary Figure S1). As a result, this study detected two independent signals through the regional plots. These SNPs of signal 1 (rs4496586) and signal 2 (rs223489) showed significantly high levels of CKD (*p* = 2.61 × 10^−3^ and *p* = 7.08 × 10^−3^), eGFR (*p* = 1.43 × 10^−9^ and *p* = 9.42 × 10^−10^), creatinine (*p* = 1.04 × 10^−8^ and *p* = 6.72 × 10^−10^), and uric acid (*p* = 1.83 × 10^−3^ and *p* = 8.61 × 10^−3^). To clearly identify secondary signals, this study performed conditional analyses, including the most significant SNPs of CKD, eGFR, creatinine, and uric acid in a stepwise manner. eGFR and creatinine found additional independent signals at the *MANBA* locus after conditioning for the most significant variants. In contrast, the conditional analyses for CKD and uric acid detected no conditionally independent signals (Supplementary Table S1).

## 4. Discussion

CKD is a complex disease caused by impaired kidney function [21]. In addition, CKD is associated with serious complications such as cardiovascular disease, hyperlipidemia, hypertension, and metabolic bone disease [22,23,24]. The prevalence of CKD reported in Korea is lower than that confirmed in other countries [3,25]. However, the early diagnosis and treatment of CKD are important in preventing various complications [26]. This study performed an association analysis of variants in the *MANBA* gene with CKD and kidney function-related traits in a Korean cohort. The study found that 11 SNPs were not only statistically significant in CKD but were also significantly associated with kidney function-related traits such as creatinine and uric acid levels (Table 2). A previous study reported that the minor A allele of rs228611 was significantly associated with the eGFR (*β* = −0.0056, *p* = 3.58 × 10^−12^) [27]. Similar to previous results, this study showed that rs228611 with a minor G allele was associated with increases in the eGFR (*β* = 0.483, *p* = 2.51 × 10^−7^) (Supplementary Table S2). Another study identified rs223489 as a candidate eGFR variant (Effect allele = A, *β* = −0.0027, *p* = 2.6 × 10^−17^) by analyzing the human ortholog in the GWAS summary statistics (eGFR) for genes associated with abnormal kidney morphology in mice [8]. Therefore, it was suggested that rs223489, which was not previously identified as important, significantly affected the eGFR. Consistent with the previous study, our results also replicated the statistically significant association of rs223489 with the eGFR (Effect allele = G, *β* = 0.599, *p* = 2.6 × 10^−10^) in Koreans. In addition, rs223489 was detected as an important signal in CKD, eGFR, creatinine, and uric acid through the regional plots (Supplementary Figure S1).

Previous studies have reported a significant association between the eGFR and genetic variants in chromosome 4 through GWAS [9,28,29]. In addition, *NFKB1* was suggested as a target gene for kidney function [27]. Unlike previous studies, Ko et al. identified candidate genes for CKD through an integrative analysis that combined CKD-associated variants and kidney eQTL results [12]. The expression of *NFKB1* gene did not show any significance in the integrative analysis, and the *MANBA* gene, which showed statistically significant co-localization between CKD-associated variants and kidney eQTL results, was proposed as a potential target gene for the GWAS variant related to kidney function. Furthermore, the study revealed that kidney function was impaired when *MANBA* gene expression was suppressed in zebrafish. Thus, increasing the expression of the *MANBA* gene might be a potential new means to treat kidney dysfunction.

Qiu et al. suggested that many diseases are cell-type-specific, rather than organ-specific [11]. Thus, they performed eQTL analysis of the renal tubules and glomeruli and identified genes that may cause kidney disease, including the *MANBA* gene. In the present study rs4496586 had the highest significance in the association analysis between CKD and variants in the *MANBA* gene. Therefore, this study analyzed the association between the rs4496586 genotype and *MANBA* gene expression in the renal tubules and glomeruli. Our results showed that the expression level of the *MANBA* gene was significantly higher in samples with a minor allele of rs4496586 (Figure 1). In brief, rs4496586 increased the expression of the *MANBA* gene and decreased the risk of CKD in patients possessing a minor allele (C). These results are consistent with those of a previous study demonstrating the association between the expression of the *MANBA* gene and renal function in vivo [12].

Interestingly, a recent study conducted mechanistic experiments for the rs6847587 variant significantly associated with *MANBA* gene expression in renal tubules using mice and cells [15]. The results showed that a reduction in *MANBA* gene expression affected not only the structure and function of lysosomes, but also the endocytosis and autophagy pathways in vivo, leading to tubular damage, inflammation activation, and fibrosis. However, the study group mentioned the limitation of not using CKD-related variants. Our results from analyzing the association between CKD and *MANBA* gene variants showed that rs6847587 significantly reduced the risk of CKD in participants with the minor allele. The presence of the rs6847587 variant with the minor allele was associated with increased *MANBA* gene expression. Thus, these results are consistent with a previous study demonstrating the association of the *MANBA* gene in the development of kidney disease.

In summary, this study focused on the *MANBA* gene and confirmed the association of genetic variants with CKD and kidney function-related traits such as eGFR, BUN, creatinine, and uric acid levels based on KoGES. The *MANBA* gene variants showed significant associations with CKD, consistent with a recent study demonstrating that *MANBA* gene variants were related to kidney function through an integrative analysis of eQTL and CKD-related GWAS results. Moreover, this study confirmed *MANBA* gene expression according to genotypes through eQTL analysis and demonstrated that the *MANBA* gene variants affecting renal tubules and glomeruli were significantly related to CKD. However, in vivo studies are needed to determine the direct effects of *MANBA* gene variants, which are highly correlated with CKD, on kidney function. Future studies also need to evaluate the association between CKD and *MANBA* gene variants through population-specific analysis.

## Figures and Tables

**Table 1:** Characteristics of participants in the Korean population.

Characteristics	Quantitative Trait Analysis	Case-Control Analysis for CKD		
		Controls	Cases	*p*-Value *
Number of participants	58,700	30,813	1130	
Gender [men (%)]	20,293 (34.57)	9170 (29.76)	528 (46.73)	<0.001
Age (M years ± SD)	53.8 ± 8.02	52.27 ± 7.53	60.92 ± 6.91	<0.001
Height (M cm ± SD)	160.72 ± 7.93	160.10 ± 7.76	161.10 ± 8.02	<0.001
Weight (M kg ± SD)	61.89 ± 9.89	60.95 ± 9.65	64.46 ± 10.09	<0.001
BMI (M kg/m^2^ ± SD)	23.67 ± 2.52	23.57 ± 2.66	24.76 ± 2.87	0.557
eGFR (mL/min/1.73 m^2^)	91.22 ± 16.54	103.35 ± 11.82	53.80 ± 9.69	<0.001
BUN (mg/dL)	14.44 ± 3.76	13.73 ± 3.54	18.87 ± 4.67	<0.001
Uric acid (mg/dL)	4.68 ± 1.25	4.38 ± 1.13	5.96 ± 1.59	<0.001
Creatinine (mg/dL)	0.80 ± 0.17	0.71 ± 0.11	1.22 ± 0.20	<0.001

* Significant differences in the characteristics between the cases and controls were determined by the Student’s *t*-test.

**Table 2:** Results of association analysis of the SNPs in the *MANBA* gene with chronic kidney disease and kidney function-related traits (*r*^2^ < 0.8).

No.	SNP	Minor Allele	MAF	Function	CKD		eGFR		Creatinine		Uric Acid	
					OR (95%CI)	*p*-Value	*β* ± S.E	*p*-Value	*β* ± S.E	*p*-Value	*β* ± S.E	*p*-Value
1	rs4496586	C	0.488	Intron	0.87 (0.80–0.95)	2.61 × 10^−3^	0.565 ± 0.093	1.43 × 10^−9^	−0.0042 ± 0.00073	1.04 × 10^−8^	−0.019 ± 0.006	1.83 × 10^−3^
2	rs223497	C	0.450	Intron	0.88 (0.81–0.97)	6.10 × 10^−3^	0.406 ± 0.094	1.55 × 10^−5^	−0.0034 ± 0.00073	3.91 × 10^−6^	−0.013 ± 0.006	0.037
3	rs223489 *	G	0.357	Intron	0.88 (0.80–0.97)	7.08 × 10^−3^	0.599 ± 0.098	9.42 × 10^−10^	−0.0047 ± 0.00076	6.72 × 10^−10^	−0.017 ± 0.006	8.61 × 10^−3^
4	rs34768739	GA	0.455	Intron	0.89 (0.81–0.97)	7.65 × 10^−3^	0.586 ± 0.094	3.86 × 10^−10^	−0.0042 ± 0.00073	9.15 × 10^−9^	−0.018 ± 0.006	3.18 × 10^−3^
5	rs34642884	GC	0.471	Intron	0.89 (0.82–0.97)	9.30 × 10^−3^	0.535 ± 0.093	1.05 × 10^−8^	−0.0039 ± 0.00073	6.87 × 10^−8^	−0.019 ± 0.006	2.07 × 10^−3^
6	rs1054037	T	0.472	Intron	0.89 (0.82–0.98)	0.012	0.530 ± 0.093	1.41 × 10^−8^	−0.0039 ± 0.00073	8.63 × 10^−8^	−0.019 ± 0.006	2.36 × 10^−3^
7	rs6847587 *	G	0.455	Intron	0.90 (0.82–0.98)	0.014	0.569 ± 0.094	1.22 × 10^−9^	−0.0041 ± 0.00073	2.46 × 10^−8^	−0.018 ± 0.006	3.13 × 10^−3^
8	rs227361 *	C	0.451	Intron	0.90 (0.82–0.98)	0.015	0.571 ± 0.094	1.13 × 10^−9^	−0.0041 ± 0.00073	2.62 × 10^−8^	−0.019 ± 0.006	2.15 × 10^−3^
9	rs228611 *	G	0.468	Intron	0.90 (0.82–0.98)	0.016	0.483 ± 0.094	2.51 × 10^−7^	−0.0035 ± 0.00073	1.85 × 10^−6^	−0.016 ± 0.006	9.43 × 10^−3^
10	rs147730991	T	0.014	Intron	1.44 (1.05–1.98)	0.022	−0.396 ± 0.394	0.315	0.0042 ± 0.00307	0.175	−0.020 ± 0.026	0.429
11	rs36126232	CT	0.402	Intron	0.90 (0.82–0.99)	0.023	0.401 ± 0.096	2.76 × 10^−5^	−0.0036 ± 0.00075	1.92 × 10^−6^	−0.016 ± 0.006	0.012
12	rs223498	C	0.453	Intron	0.91 (0.83–0.99)	0.034	0.389 ± 0.094	3.49 × 10^−5^	−0.0030 ± 0.00073	3.14 × 10^−5^	−0.010 ± 0.006	0.111
13	rs223487	C	0.254	Intron	0.90 (0.81–0.99)	0.035	0.616 ± 0.107	9.73 × 10^−9^	−0.0045 ± 0.00084	7.10 × 10^−8^	−0.017 ± 0.007	0.016
14	rs11097790	C	0.384	Intron	0.91 (0.83–0.99)	0.038	0.432 ± 0.097	7.66 × 10^−6^	−0.0029 ± 0.00075	9.06 × 10^−5^	−0.012 ± 0.006	0.055
15	rs12650217	C	0.4637	Intron	0.92 (0.84–1.00)	0.054	0.355 ± 0.094	1.50 × 10^−4^	−0.0028 ± 0.00073	1.46 × 10^−4^	−0.0099 ± 0.0061	0.106
16	rs78905355	C	0.01218	Intron	0.69 (0.46–1.05)	0.084	1.586 ± 0.423	1.80 × 10^−4^	−0.0123 ± 0.00330	1.92 × 10^−4^	−0.0220 ± 0.0277	0.427
17	rs11454438	CA	0.4807	Intron	1.08 (0.99–1.17)	0.102	−0.400 ± 0.094	1.92 × 10^−5^	0.0029 ± 0.00073	6.02 × 10^−5^	0.0123 ± 0.0061	0.044
18	rs223503	G	0.2466	Intron	0.93 (0.84–1.03)	0.185	0.477 ± 0.108	1.08 × 10^−5^	−0.0032 ± 0.00084	1.36 × 10^−4^	−0.0099 ± 0.0071	0.164
19	rs170563 *	C	0.2613	Intron	0.94 (0.85–1.04)	0.213	0.480 ± 0.107	6.92 × 10^−6^	−0.0033 ± 0.00083	7.33 × 10^−5^	−0.0110 ± 0.0070	0.116
20	rs227374	A	0.2572	Intron	0.95 (0.86–1.05)	0.321	0.459 ± 0.107	1.86 × 10^−5^	−0.0032 ± 0.00083	1.52 × 10^−4^	−0.0108 ± 0.0070	0.123

SNP, single nucleotide polymorphism; MAF, minor allele frequency; CKD, chronic kidney disease; eGFR, estimated glomerular filtration rate; *β*, regression coefficient; S.E, standard error; OR, odds ratio; CI, confidence interval. eGFR, creatinine, and uric acid levels used in the linear regression were adjusted for age and gender. Odds ratios were calculated after adjusting for age and gender. Both logistic and linear regressions were conducted using an additive model. * SNPs replicated in the kidney-related results from other studies.

## Data Availability

The data presented in this study are available on request from the corresponding author. The data are not publicly available due to ethnical concerns.

## Supplementary Materials

The following are available online at https://www.mdpi.com/article/10.3390/jcm10112255/s1, Table S1: Conditional analyses of multiple SNPs at the *MANBA* locus. Table S2: Results of an association analysis of the SNPs in the *MANBA* gene with chronic kidney disease and kidney function-related traits. Figure S1: Regional plots of the association results of *MANBA* SNPs with CKD (a) and kidney function-related traits such as eGFR (b), creatinine (c), and uric acid levels (d) in the HEXA cohort. The statistical significance (−log_10_ *p*-value) of the analyzed SNPs is plotted. The colors indicate the linkage disequilibrium (r^2^) between rs223489 and the remaining SNPs. The genetic recombination rates are shown on the right *y*-axis. The plots were generated by the LocusZoom program (http://csg.sph.umich.edu/locuszoom/ (accessed on 13 April 2021)).
