# Supplementary material for: Association between MANBA Gene Variants and Chronic Kidney Disease in a Korean Population
Source: J Clin Med. 2021 May 23;10(11):2255. doi: 10.3390/jcm10112255 (PMC8196967; doi:10.3390/jcm10112255)
Supplement: Supplementary file 1 [file jcm-10-02255-s001.zip › Suppelmetary Figure S1.pdf]

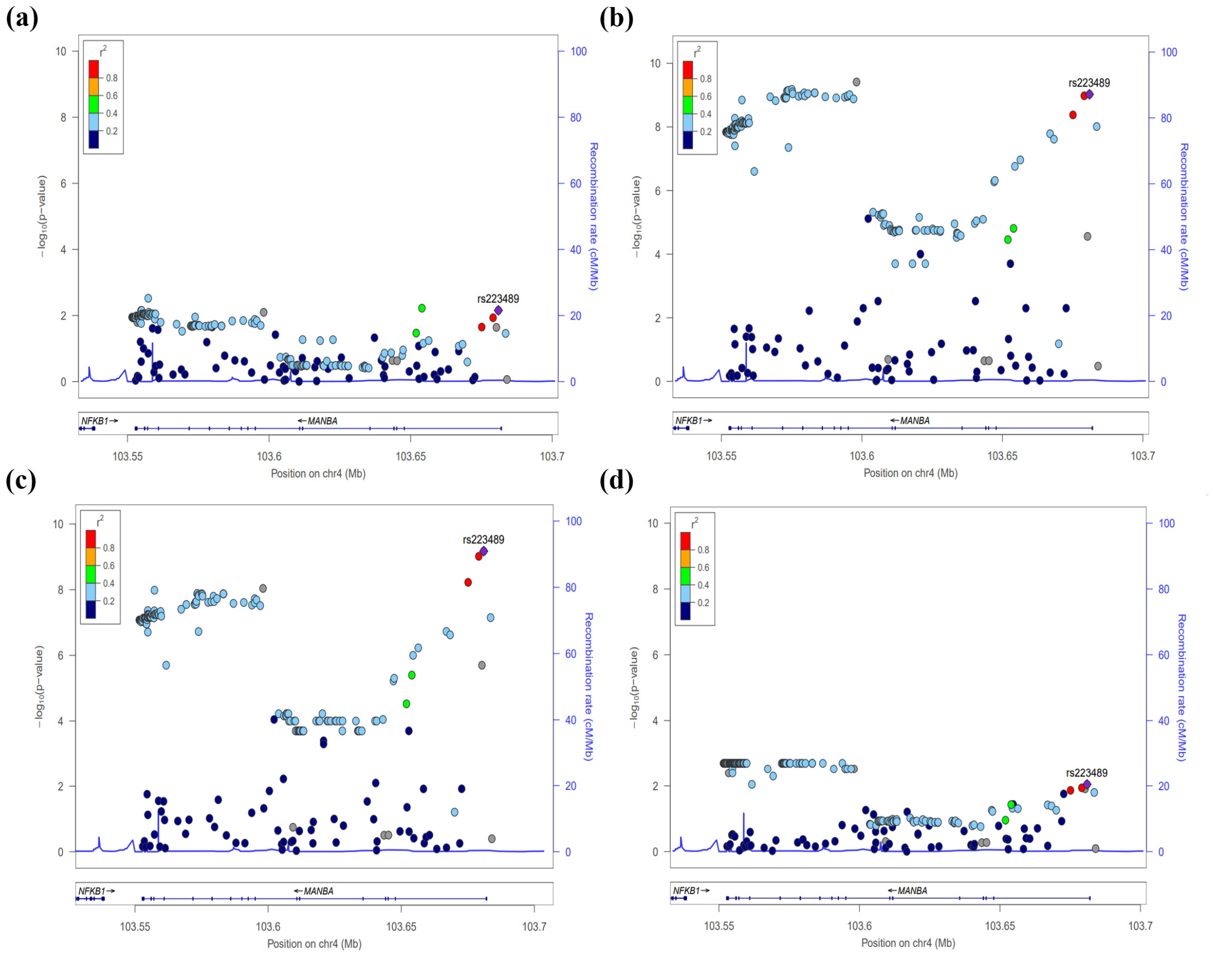

Figure S1. Regional plots of the association results of *MANBA* SNPs with CKD (**a**) and kidney function-related traits such as eGFR (**b**), creatinine (**c**), and uric acid levels (**d**) in the HEXA cohort. The statistical significance ($-\log_{10}$ *p*-value) of the analyzed SNPs is plotted. The colors indicate the linkage disequilibrium (r^2) between rs223489 and the remaining SNPs. The genetic recombination rates are shown on the right *y*-axis. The plots were generated by the LocusZoom program (http://csg.sph.umich.edu/locuszoom/ (accessed on 13 April 2021)).
